# Novel biomarkers that assist in accurate discrimination of squamous cell carcinoma from adenocarcinoma of the lung

**DOI:** 10.1186/s12885-016-2792-1

**Published:** 2016-09-29

**Authors:** Kazuya Takamochi, Hiroko Ohmiya, Masayoshi Itoh, Kaoru Mogushi, Tsuyoshi Saito, Kieko Hara, Keiko Mitani, Yasushi Kogo, Yasunari Yamanaka, Jun Kawai, Yoshihide Hayashizaki, Shiaki Oh, Kenji Suzuki, Hideya Kawaji

**Affiliations:** 1 Department of General Thoracic Surgery, Juntendo University School of Medicine, 1-3, Hongo 3-chome, Bunkyo-ku, Tokyo, 113-8431 Japan; 2 Preventive Medicine and Applied Genomics Unit, RIKEN Advanced Center for Computing and Communication, 1-7-22 Suehiro-cho, Tsurumi-ku, 230-0045 Yokohama, Japan; 3 RIKEN Preventive Medicine and Diagnosis Innovation Program, 2-1 Hirosawa, Wako-shi, Saitama 351-0198 Japan; 4 Center for Genomic and Regenerative Medicine, Juntendo University School of Medicine, 1-3, Hongo 3-chome, Bunkyo-ku, Tokyo, 113-8431 Japan; 5 Department of Human Pathology, Juntendo University School of Medicine, 1-3, Hongo 3-chome, Bunkyo-ku, Tokyo, 113-8431 Japan

## Abstract

**Background:**

Targeted therapies based on the molecular and histological features of cancer types are becoming standard practice. The most effective regimen in lung cancers is different between squamous cell carcinoma (SCC) and adenocarcinoma (AD). Therefore a precise diagnosis is crucial, but this has been difficult, particularly for poorly differentiated SCC (PDSCC) and AD without a lepidic growth component (non-lepidic AD). Biomarkers enabling a precise diagnosis are therefore urgently needed.

**Methods:**

Cap Analysis of Gene Expression (CAGE) is a method used to quantify promoter activities across the whole genome by determining the 5’ ends of capped RNA molecules with next-generation sequencing. We performed CAGE on 97 frozen tissues from surgically resected lung cancers (22 SCC and 75 AD), and confirmed the findings by immunohistochemical analysis (IHC) in an independent group (29 SCC and 45 AD).

**Results:**

Using the genome-wide promoter activity profiles, we confirmed that the expression of known molecular markers used in IHC for SCC (CK5, CK6, p40 and desmoglein-3) and AD (TTF-1 and napsin A) were different between SCC and AD. We identified two novel marker candidates, SPATS2 for SCC and ST6GALNAC1 for AD, as showing comparable performance and complementary utility to the known markers in discriminating PDSCC and non-lepidic AD. We subsequently confirmed their utility at the protein level by IHC in an independent group.

**Conclusions:**

We identified two genes, SPATS2 and ST6GALNAC1, as novel complemental biomarkers discriminating SCC and AD. These findings will contribute to a more accurate diagnosis of NSCLC, which is crucial for precision medicine for lung cancer.

**Electronic supplementary material:**

The online version of this article (doi:10.1186/s12885-016-2792-1) contains supplementary material, which is available to authorized users.

## Background

Non-small cell lung cancers (NSCLCs) account for approximately 89 % of all lung cancers. NSCLCs are further classified into adenocarcinoma (AD: 45 %), squamous cell carcinoma (SCC: 24 %), and large cell carcinomas (3 %), respectively [1]. Recent developments in targeted therapies, such as pemetrexed [2] and bevacizumab [3, 4], require precise typing of NSCLCs, since they are inappropriate for SCC. Accurate discrimination of SCC from the remaining NSCLCs is crucial for choosing the appropriate treatment regimen.

SCC is defined as a malignant epithelial tumor showing keratinization and/or intercellular bridges. These features are evident in well differentiated (WD) tumors; however, they are only focally present in poorly differentiated (PD) tumors. The histological diagnosis of SCC is sometimes difficult for PD tumors based on small biopsy or cytology samples [5, 6]. AD is conventionally diagnosed based on the histological characteristics of luminal formation and/or intracytoplasmic mucin in the tumor. About 90 % of lung ADs consist of mixed heterogeneous components, such as lepidic, acinar, papillary, solid and micropapillary components, where the lepidic component is easy to obtain as a well preserved tissue structure compared to the other components because it is usually observed in the peripheral area of the tumor. If a lepidic component is found in a diagnostic material, it is easy to diagnose an AD. However, if a tumor biopsy specimen does not have a lepidic component, the histological diagnosis of AD is sometimes difficult based on small biopsy or cytology samples, especially when the tissue structure is not preserved. In particular, discriminating between PDSCC and solid predominant AD is challenging to the pathologists based solely on the morphological findings of tumors [5, 6].

Cellular function is implemented with a series of molecules produced by the cell. Distinct types of cells can be discriminated at the molecular level even if they are similar to each other morphologically. The emergence of next-generation sequencing technologies enabled us to obtain accurate snapshot molecules, in particular DNA and RNA. Cap Analysis Gene Expression (CAGE) is a genome-wide approach to sequencing only the 5’-ends of capped RNAs [7], and its profiles represent promoter activities based on the frequencies of transcription starting sites (TSSs). CAGE was used to annotate functional elements within the human genome in the ENCODE project [8], and it was used to monitor global transcriptome states characterizing diverse cell types across the human body in the FANTOM5 project [8–10]. Obtaining an accurate map of transcriptome in a wide range of primary cells, organs, and cell lines enabled us to understand a series of observations, such as structural relationships between cancer cell lines [11], mesothelial signatures in high-grade serous ovarian cancer [12], and regulatory regions of the three genes involved in Rett Syndrome [13].

The present study is the first use of CAGE to survey primary tumors for a specific clinical problem, in this case, the identification of biomarkers enabling a precise diagnosis of SCC and AD. Our genome-wide survey led us to identify two novel markers that complement known markers to recognize a unique set of tumors. Follow-up experiments on another group of patients confirmed their performance for discriminating SCC from AD.

## Methods

### Patients enrolled for biomarker exploration by CAGE: The discovery set

The sample collection was conducted at Juntendo University in Japan, between February 2010 and January 2011. Under a protocol approved by the institutional review board of Juntendo University (No.2012069), 97 tumor tissue specimens were collected after the tissue donors provided written informed consent. In the operating room, 3–5 mm^3^ cubes of fresh lung cancer tissue were dissected and immediately placed in 1.0 ml of RNAlater RNA Stabilization Reagent (Qiagen, GmbH, Germany, Hilden) for 24–48 h at 4 °C for RNA stabilization. Thereafter, the specimens were stored at −80 °C until RNA extraction. Total RNA was extracted from the frozen tissue sections according to the standard protocol.

The gold standard of histological diagnosis used in the present study is based on the permanent pathological reports made by at least two experienced pathologists in accordance with the 2004 WHO Classification of Lung Tumors [14]. In clinical practice, pathologists make diagnoses based on histological criteria (presence of a malignant epithelial tumor showing keratinization and/or intercellular bridges for SCC and the presence of luminal formation and/or intracytoplasmic mucin in the tumor for AD). Immunohistochemical analysis (IHC) such as TTF-1 or p40 is performed only in cases where a definitive diagnosis is difficult based solely on the above-mentioned histological criteria. If no morphological features specific to SCC or AD were noted, tumors were diagnosed as large cell carcinoma, and the patient was excluded from the study cohort.

ADs were further subtyped into three groups based on the lepidic growth component in each tumor: pure lepidic AD, AD with a 100 % lepidic growth component; mixed lepidic AD, AD with any lepidic component and non-lepidic AD, AD without a lepidic component. SCCs were also subtyped into three groups based on the degree of keratinization and/or intercellular bridges: WDSCC, moderately differentiated (MD) SCC and PDSCC. The 97 frozen tumor tissues consists of 22 SCC and 75 AD, including five cases of WDSCC, 14 MDSCC, three PDSCC, seven pure lepidic AD, 56 mixed lepidic AD, and 12 cases of non-lepidic AD.

### Patients enrolled for biomarker validation by an IHC: The validation set

In addition to the collection above, 74 tumors were collected by surgical resection of lung cancers (SCC, *n* = 29; AD, *n* = 45) at Juntendo University between February 2013 and November 2013 under the same protocol described above. The 74 tumors consisted of four WDSCC, 14 MDSCC, 11 PDSCC, seven pure lepidic AD, 22 mixed lepidic AD, and 16 non-lepidic AD, which were pathologically diagnosed using the same criteria as the samples collected for the CAGE analysis.

### CAGE assay

CAGE libraries were prepared following the previously described protocol [15]. In brief, the total RNA extracts were subjected to a reverse transcription reaction with SuperScript III (Life Technologies, Carlsbad, CA, USA). After purification using RNAclean XP (Beckman Coulter, Brea, CA, USA), double stranded-RNA/cDNA were oxidized with sodium periodate to generate aldehydes from the diols of the ribose at the cap structure and 3’-end, and these were biotinylated with biotin hydrazide (Vector Laboratories, Burlingame, CA, USA). The remaining single-stranded RNA was digested with RNase I (Promega, Madison, WI, USA) before capturing the biotinylated cap structure with magnetic streptavidin beads (Dynal Streptavidin M-270; Life Technologies, Carlsbad, CA, USA). Single-stranded cDNA was recovered by heat denaturation, and was ligated with the 3’-end and 5’-end adaptors specific to the samples, subsequently. Double-stranded cDNAs were prepared by using a primer and DeepVent (exo^−^) DNA polymerase (New England, Ipswich, MA, USA), and were mixed so that sequencing with one lane could produce data from eight samples. Three nanograms of the mixed samples were used to prepare 120 μl of loading sample [15], which was loaded on c-Bot, and sequenced by an Illumina HiSeq2500 sequencer (Illumina, San Diego, CA, USA).

### Computational analysis of CAGE data to identify candidate markers

The original samples from which individual reads were obtained were identified with the ligated adaptor sequences. After discarding reads including a base ‘N’ or that hit a ribosomal RNA sequence (U13369.1) with rRNAdust [16], the reads were aligned to the reference genome (hg19) using BWA (version 0.7.10) [17], where poorly aligned reads (mapping quality < 20) were discarded using SAMtools (version 0.1.19) [18]. Only libraries with more than two million mapped reads were used for further analyses. The robust peak set [9] was used as a reference set for TSS regions, and the number of mapped reads starting from these regions were used as raw signals for the promoter activities. Inactive TSS regions, with counts per million (CPM) ≤ 1 in more than 77 % of the samples in both subtypes, were filtered out [19], and 46,238 regions remained for the downstream analysis. Multi-dimensional scaling (MDS) and differential analyses were conducted using the edgeR (version 2.6.7) [20] in R/bioconductor [21].

### IHC

Four μm-thick tissue sections were prepared from formalin-fixed paraffin-embedded blocks and subjected to IHC. The antibodies used and their conditions are described in Additional file 1: Table S1. IHC staining was performed using an Envision Kit (Dako, Grostrup, Denmark) with substrate-chromogen solution. A glass slide was visually inspected and scored as follows for novel markers identified by CAGE: score 0, no tumor cells showing immunoreactivity; score 2, more than 50 % of tumor cells showing moderate or more severe immunoreactivity; and score 1, not classified as score 0 or 2. Existing IHC markers, such as TTF-1, napsin A, p40, cytokeratin (CK) 5, CK6, and desmoglein-3 (DSG3), were scored as follows: score 0, no tumor cells showing immunoreactivity; score 1, less than 10 % of tumor cells showing immunoreactivity; and score 2, 10 % or more of tumor cells showing immunoreactivity.

Scores of 0 and 1 were considered negative, and a score of 2 was considered positive. The scoring was performed by two independent pathologists (authors T.S. and K.H.) without prior knowledge of the clinicopathological data, and discrepancies were resolved by re-evaluation to reach a consensus.

### Clustering of tumors based on the IHC results

The distances between the samples with the IHC-based marker expression patterns were calculated as Euclidean distances for the positive/negative state, where the state was assigned as 1 (positive) when the IHC score was 2, and was assigned as 0 (negative) otherwise. The average linkage clustering was performed independently on the discovery set and validation sets, by using R (version 3.0.2, http://www.r-project.org/),

## Results

### Quantitative profiles of genome-wide promoter activities in lung cancer

**Fig. 1:**
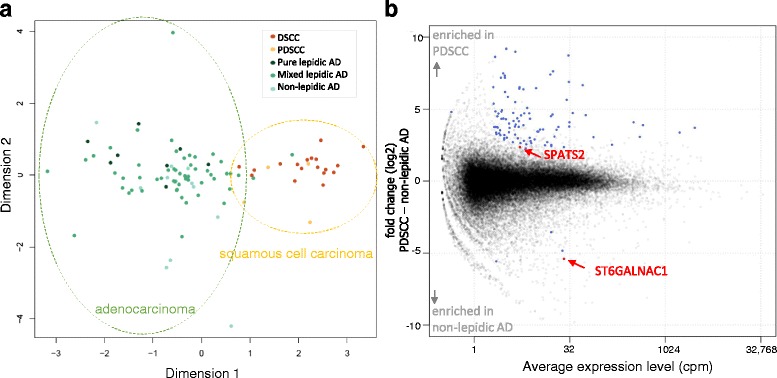
Promoter activities in lung cancer. (**a**) An MDS plot. Similarities (distances) between individual carcinomas in the space of promoter activities (CAGE profiles) are visualized in two dimensions by the multi-dimensional scaling implemented in the edgeR [20], where individual dots represent individual carcinomas and similar carcinomas are plotted closely. The dot colors represent carcinoma subtypes as indicated in the legend, and the dotted line indicates groups of carcinomas. (**b**) An MA-plot of the differential analysis between PDSCC and non-lepidic AD. The X-axis represents the average expression levels in cpm, and the Y-axis represents the fold-changes in the log2 scale. Individual dots represent the activities of individual promoters, and the blue dots indicate promoters with statistically significant differences (fold-change > 4, CPM > 4 and FDR < 0.01), and the red dots indicate the marker candidates we selected

We obtained quantitative promoter activity profiles from 97 lung cancer tissues, consisting of 75 AD and 22 SCC, using a CAGE protocol [7] with a next generation sequencer (HiSeq2500). The two types of carcinoma are known to show different expression patterns [22], which were confirmed in our CAGE data (Fig. 1a). We also found that several cases were not clearly separated, which is consistent with previous studies using microarrays [22] or IHC [23]. In particular, PDSCC and non-lepidic AD are difficult to be distinguished in the clinical setting when relying on protein markers such as napsin A [24, 25] and TTF-1 [24, 25] (AD markers), or p40 [26, 27], DSG3 [24, 28], CK5 [24, 25] and CK6 [25] (SCC markers).

### SPATS2 and ST6GALNAC1 discriminate PDSCC and non-lepidic AD

We focused on the two difficult to distinguish subtypes, PDSCC and non-lepidic AD. Of 65 differentially expressed promoters with (i) statistical significance (FDR < 0.01), (ii) a high fold-change (>4-fold), and (iii) substantial expression (>4 cpm), 62 of them were highly expressed in PDSCC and three were highly expressed in non-lepidic AD (Fig. 1b, blue and red dots). We found that seven promoters distinguished the subtypes completely after setting a threshold, and we manually selected two promoters corresponding to protein-coding genes: spermatogenesis associated, serine-rich 2 (SPATS2) [29] and ST6 (alpha-N-acetyl-neuraminyl-2,3-beta-galactosyl-1,3)-N-acetylgalactosaminide alpha-2,6-sialyltransferase 1 (ST6GALNAC1) [30], as candidate biomarkers (Fig. 1b, red dots).

**Fig. 2:**
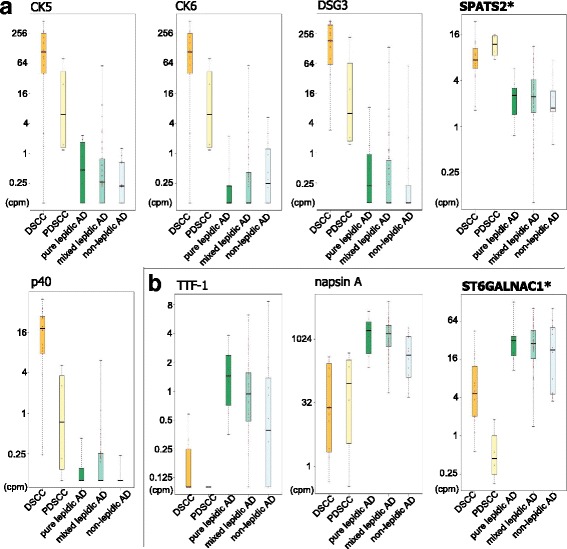
Promoter activity levels of known markers and novel candidates. (**a**) The promoter activities of known markers for AD and the novel candidate are shown in boxplots based on the carcinoma subtypes. (**b**) Equivalent boxplots for known markers of SCC and the candidate

As shown in Fig. 2a, SPATS2 was active in SCC, particularly PDSCC, and less active in AD overall. Notably, it was more active in PDSCC than differentiated SCC (DSCC), which is unique for this molecule. In contrast, ST6GALNAC1 was almost absent only in PDSCC (<1 cpm; Fig. 2b). TTF-1, one of the known AD markers, was absent in PDSCC, but was also often absent in some of the non-lepidic AD cases. While napsin A is another AD marker, it was often active in some of the PDSCC cases. We found that both of SPATS2 and ST6GALNAC1 showed unique expression patterns not found for the known markers.

### IHC identified the proteins of the candidate marker genes in tumor tissues

**Fig. 3:**
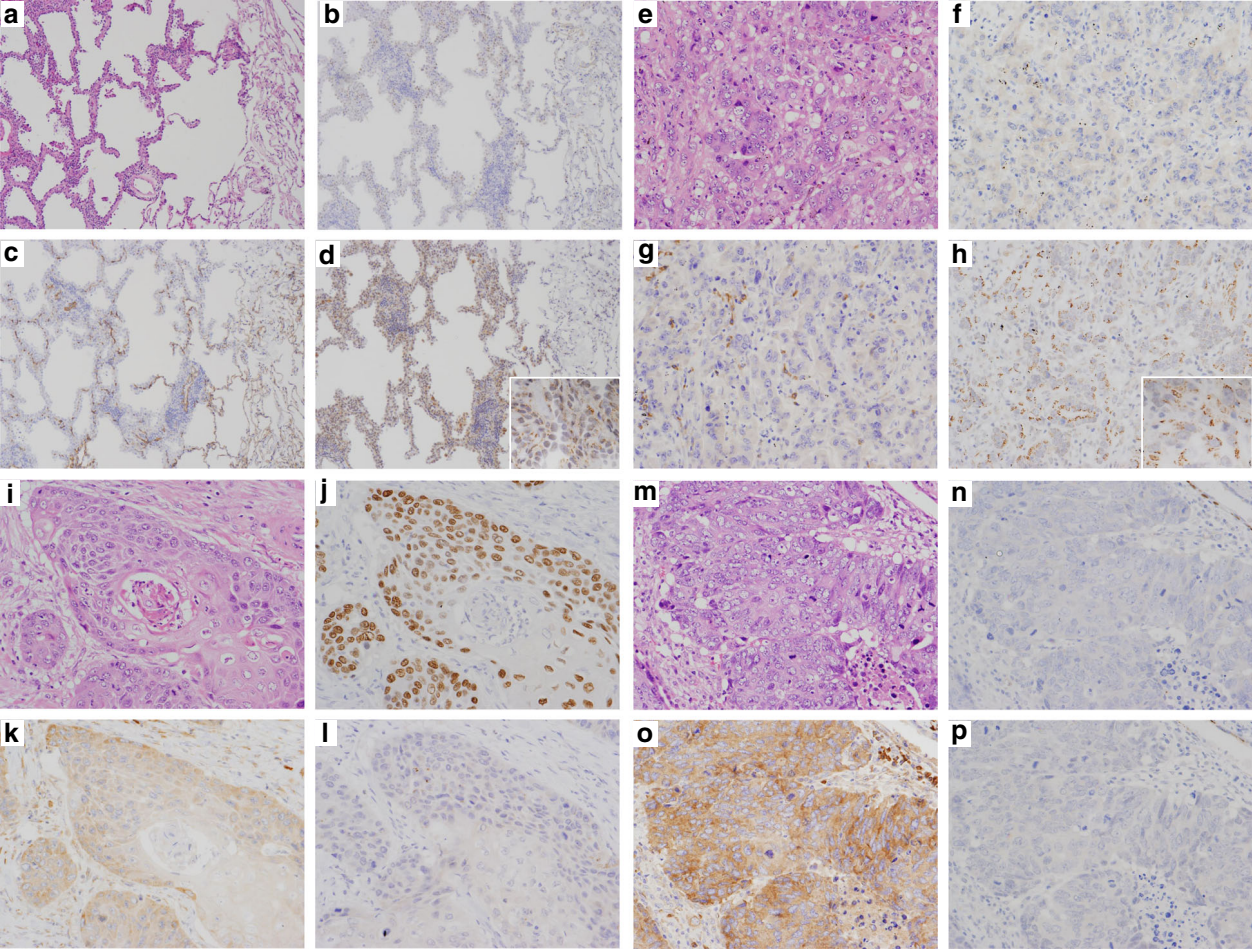
IHC for the novel marker candidates. A case of pure lepidic AD (**a**-**d**). H.E. staining (**a**) and IHC for TTF-1 (**b**), SPATS2 (**c**) and ST6GALNAC1 (**d**). The tumor cells are diffusely positive for ST6GALNAC1, but negative for TTF1 and SPATS2. A case of non-lepidic AD (**e**-**h**). H.E. staining (**e**) and IHC for TTF-1 (**f**), SPATS2 (**g**) and ST6GALNAC1 (**h**). The tumor cells are diffusely positive for ST6GALNAC1, but negative for TTF1 and SPATS2. Note that infiltrating plasma cells are also positive for SPATS2 (**g**). A case of WDSCC (**i**-**l**). H.E. staining (**i**) and IHC for p40 (**j**), SPATS2 (**k**) and ST6GALNAC1 (**l**). The tumor cells are diffusely positive for SPATS2 and p40, but negative for ST6GALNAC1. A case of PDSCC (**m**-**p**). H.E. staining (**m**) and IHC for p40 (**n**), SPATS2 (**o**) and ST6GALNAC1 (**p**). The tumor cells are diffusely positive for SPATS2, but negative for p40 and ST6GALNAC1. Note that SPATS2 staining is more sensitive than p40 staining. (original magnifications: x100, insets: x400)

We next examined the candidate biomarkers at the protein level. We performed an IHC analysis on paraffin-embedded tumors obtained from the same patients analyzed by CAGE above, and found clear contrasts between the staining patterns of SPATS2 and ST6GALNAC1 between AD and SCC, even in the PD form of each tumor type (Fig. 3). ST6GALNAC1 was more sensitive than TTF-1 in some non-lepidic AD cases (Fig. 3f, h), and SPATS2 was more sensitive than p40 in some PDSCC cases (Fig. 3n, o). Notably, SPATS2 was localized to the cytoplasm of tumor cells, although we also found positive staining at the basal membrane of the alveolar septum and infiltrating plasma cells. ST6GALNAC1 was localized on the cellular membrane of tumor cells but also stained with bronchial epithelium.

### Significant contribution to discriminating the two subtypes

**Fig. 4:**
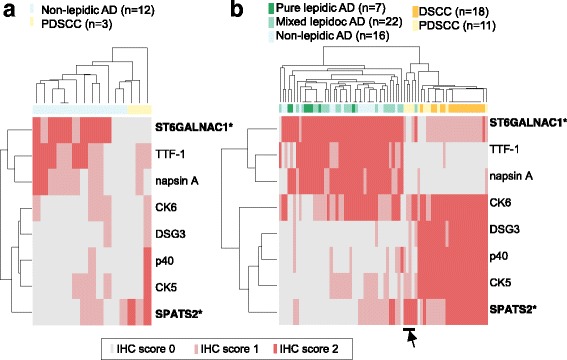
The results of IHC with the novel and known markers. (**a**) The presence of the known markers and the candidate markers was examined by IHC of carcinoma tissues of non-lepidic AD and PDSCC obtained from the same patients evaluated in the CAGE analysis. The staining patterns are scored (IHC score 0, 1, and 2) as described in the METHODS section, and the scores are visualized as heatmaps, where the tissues and markers are clustered based on the IHC scores. (**b**) Equivalent heatmaps based on the results of an independent group of patients, consisting of pure lepidic AD and mixed lepidic AD, DSCC, as well as non-lepidic AD and PDSCC

We then examined the performance of the new markers in comparison with the existing markers by IHC. Paraffin-embedded tumors obtained from the same patients used in the CAGE analysis were immunostained for the six known markers, as well as two novel marker candidates. The heatmap showing the staining scores (Fig. 4a) indicated that all of the markers were reasonably useful in discriminating the two subtypes. Notably, SPATS2 and ST6GALNAC1 were more sensitive for PDSCC and non-lepidic AD (~66 %) than the existing markers respectively when taking an IHC score of 2 as positive (Table 1).

**Table 1:** Evaluation of the markers using the discovery set with 12 non-lepidic AD and three PDSCC patients

AD markers	(Marker status)	(+)		(−)		Sensitivity	Specificity	PPV	NPV	Accuracy
	(subtype)	AD	SCC	AD	SCC	(95 % CI†)	(95 % CI)	(95 % CI)	(95 % CI)	(95 % CI)
	ST6GALNAC1*	8	0	4	3	0.667 (0.349–0.901)	1.000 (0.292–1.000)	1.000 (0.631–1.000)	0.429 (0.099–0.816)	0.733 (0.099–0.816)
	TTF-1	5	0	7	3	0.417 (0.152–0.723)	1.000 (0.292–1.000)	1.000 (0.478–1.000)	0.300 (0.067–0.652)	0.533 (0.266–0.787)
	napsin A	2	0	10	3	0.167 (0.021–0.484)	1.000 (0.292–1.000)	1.000 (0.158–1.000)	0.231 (0.050–0.538)	0.333 (0.118–0.616)
SCC markers	(Marker status)	(+)		(−)		Sensitivity	Specificity	PPV	NPV	Accuracy
	(subtype)	SCC	AD	SCC	AD	(95 % CI)	(95 % CI)	(95 % CI)	(95 % CI)	(95 % CI)
	SPATS2*	2	0	1	12	0.667 (0.094–0.992)	1.000 (0.735–1.000)	1.000 (0.158–1.000)	0.923 (0.640–0.998)	0.933 (0.681–0.998)
	CK5	1	0	2	12	0.333 (0.008–0.906)	1.000 (0.735–1.000)	1.000 (0.025–1.000)	0.857 (0.572–0.982)	0.867 (0.595–0.983)
	DSG3	0	0	3	12	0.000 (0.000–0.708)	1.000 (0.735–1.000)	N.A.	0.800 (0.519–0.957)	0.800 (0.519–0.957)
	p40	1	0	2	12	0.333 (0.008–0.906)	1.000 (0.735–1.000)	1.000 (0.025–1.000)	0.857 (0.572–0.982)	0.867 (0.595–0.983)
	CK6	0	0	3	12	0.000 (0.000–0.708)	1.000 (0.735–1.000)	N.A.	0.800 (0.519–0.957)	0.800 (0.519–0.957)

*PPV* Positive predictive value, *NPV* Negative predictive value, *95 % CI* 95 % confidence interval, *N.A* Not available

†:95 % CIs of sensitivity, specificity, PPV, NPV and accuracy were estimated by the Clopper-Pearson method

* Novel biomarkers identified in the present study

### Validation with an independent group of patients confirmed the performance of the novel markers

We further assessed their performance of these markers with an independent group of patients, consisting of 16 non-lepidic AD and 11 PDSCC. We confirmed the above results, with the highest sensitivity and accuracy being for these two markers (Table 2). We further expanded the validation group by including seven cases of pure lepidic AD, 22 mixed lepidic AD, four WDSCC and 14 MDSCC (in total, *n* = 74), and confirmed that ST6GALNAC1 had the highest sensitivity for detecting of any type of AD (Additional file 1: Table S2). We also confirmed the unique SPATS2 staining pattern, where a specific subgroup of PDSCC (indicated by the arrowhead in Fig. 4b) was not detectable without SPATS2.

**Table 2:** Evaluation of the markers using the validation set with 16 non-lepidic AD and 11 PDSCC patients

AD markers	(Marker status)	(+)		(−)		Sensitivity	Specificity	PPV	NPV	Accuracy
	(subtype)	AD	SCC	AD	SCC	(95 % CI†)	(95 % CI)	(95 % CI)	(95 % CI)	(95 % CI)
	ST6GALNAC1*	15	0	1	11	0.938 (0.698–0.998)	1.000 (0.715–1.000)	1.000 (0.782–1.000)	0.917 (0.615–0.998)	0.963 (0.810–0.999)
	TTF-1	10	0	6	11	0.625 (0.354–0.848)	1.000 (0.715–1.000)	1.000 (0.692–1.000)	0.647 (0.383–0.858)	0.778 (0.577–0.914)
	napsin A	12	0	4	11	0.750 (0.476–0.927)	1.000 (0.715–1.000)	1.000 (0.735–1.000)	0.733 (0.449–0.922)	0.852 (0.663–0.958)
SCC markers	(Marker status)	(+)		(−)		Sensitivity	Specificity	PPV	NPV	Accuracy
	(subtype)	SCC	AD	SCC	AD	(95 % CI)	(95 % CI)	(95 % CI)	(95 % CI)	(95 % CI)
	SPATS2*	7	0	4	16	0.636 (0.308–0.891)	1.000 (0.794–1.000)	1.000 (0.590–1.000)	0.800 (0.563–0.943)	0.852 (0.663–0.958)
	CK5	7	0	4	16	0.636 (0.308–0.891)	1.000 (0.794–1.000)	1.000 (0.590–1.000)	0.800 (0.563–0.943)	0.852 (0.663–0.958)
	DSG3	6	0	5	16	0.545 (0.234–0.833)	1.000 (0.794–1.000)	1.000 (0.541–1.000)	0.762 (0.528–0.918)	0.815 (0.619–0.937)
	p40	7	0	4	16	0.636 (0.308–0.891)	1.000 (0.794–1.000)	1.000 (0.590–1.000)	0.800 (0.563–0.943)	0.852 (0.663–0.958)
	CK6	5	9	6	7	0.455 (0.167–0.766)	0.438 (0.198–0.701)	0.357 (0.128–0.649)	0.538 (0.251–0.808)	0.444 (0.255–0.647)

*PPV* Positive predictive value, *NPV* Negative predictive value, *95 % CI* 95 % confidence interval

†: 95 % CIs of sensitivity, specificity, PPV, NPV and accuracy were estimated by the Clopper-Pearson method

* Novel biomarkers identified in the present study

Finally, we examined the results by assuming a definitive diagnosis, rather than a diagnosis by exclusion. Additional file 1: Table S3 indicates the results of the definitive diagnosis using all combinations of a minimum number (two) of molecular markers. It showed that the combination of ST6GALNAC1 for AD and CK5 for SCC provided a definitive diagnosis at the highest accuracy, while some cases (*n* = 7, consisting of two AD and five SCC cases) remained to be unclassifiable. The unclassifiable cases were further examined (Additional file 1: Table S4), and we found that TTF-1 and SPATS2 contributed to their successful classification. Both of the novel markers are crucial for obtaining a definitive diagnosis while avoiding inconclusive cases.

## Discussion

The 2015 WHO Classification of Lung Tumors was recently published [31]. IHC markers such as p40 and TTF-1 are recommended for definitive histological diagnosis of SCC and AD when diagnosis is inconclusive based solely on the morphological features, in order to minimize the category NSCLC-not otherwise specified or large cell carcinoma.

Several IHC markers have been used for subtyping NSCLC. Most markers were identified without consideration of the histological diversity in SCC and AD, which makes their subtyping keep challenging in the clinical settings. Diagnosing of DSCC and lepidic AD is straightforward morphologically, and IHC staining is not required for a diagnosis in most of these cases. Molecular markers to discriminate subtypes that are difficult to diagnose, such as PDSCC and non-lepidic AD, have a high impact in the clinical settings. Therefore, we started our analysis to identify marker candidates based on a comparison of these subtypes.

Our genome-wide screening of promoter activities identified two marker candidates, SPATS2 as a PDSCC marker and ST6GALNAC1 as a non-lepidic AD marker. Their expression levels in individual histological subtypes suggests that they will have utility in broadly discriminating between SCC and AD, regardless of the histological diversity. CAGE was somewhat effective in this screening step, owing to its coverage of targets, namely all TSSs across the genome, and its ability to quantify precise expression levels. Although IHC is commonly used in clinical practice, its lack of these features makes it unsuitable for screening. However, one of the drawbacks in transcriptome analysis, including CAGE, of solid tissue is that the profiling target consists of heterogenous cells. In the present study, the profiled tissues likely consist of cancer cells and normal pneumocytes. While the cancerous part was obtained from a collection of samples, the resulting data requires careful interpretation. We found the largest variance in sample ranges from SCC to AD (Fig. 1), suggesting that the ratio of normal pneumocytes was not very different in the profiled tissues and has negligible impact to the CAGE profile in comparison with the difference between DSCC and AD. We decided to perform further examination based on IHC scores below, which clarify whether the potential markers represent molecular states of cancer cells or normal ones.

For a clinical diagnosis, IHC has been used more often than RNA quantification. Therefore, we asked whether the protein-level expression of these genes would also be effective for obtaining a precise diagnosis. The staining patterns with IHC were also clearly different for the subtypes. The IHC scoring of the discovery set, the group of tumors profiled by CAGE, demonstrated high sensitivities as discrimination markers (Fig. 4a, Table 1). These results not only validated the findings for the RNA expression, but also demonstrated that these genes can be used as biomarkers at either the mRNA or protein level.

Finally, we examined their diagnostic utility by using an independent set of 74 cases by IHC. ST6GALNAC1 showed higher sensitivity and accuracy than the existing IHC markers for AD, such as TTF-1 and napsin A. In contrast, SPATS2 showed a unique staining pattern, where it was positive in SCC cases, even when the staining results of the existing SCC markers (p40, DSG3, CK5 and CK6) were negative (Fig. 4b, Additional file 1: Table S2). All of these results are consistent with those of the discovery set, and confirmed their performance as their diagnosis markers in another set of tumors.

We subsequently examined the potential of these markers for obtaining a definitive diagnosis. A combination of ST6GALNAC1 for AD and CK5 for SCC had the best performance (90.5 % accuracy), while a few cases remained as inconclusive (9.5 %) (Additional file 1: Table S3). Within the inconclusive cases, a combination of TTF-1 for AD and SPATS2 for SCC provided the best performance (100 % accuracy) (Additional file 1: Table S4). In contrast, the combination of TTF-1 and p40, broadly considered to be most reliable for the differential diagnosis between SCC and AD [27, 32, 33], showed an accuracy of 77 % in our study population. These results demonstrate that the two novel makers are effective in combination with some known markers for obtaining a definitive diagnosis. A promising approach for definitive diagnosis is to perform IHC on both ST6GALNAC1 and CK5 at the first step, and then to examine both TTF-1 and SPATS2 only when the results of the first step are inconclusive.

*ST6GALNAC1* is a member of the sialyltransferase family of molecules, which was reported as overexpressed in several cancers, including gastric cancer, and as correlated with cancer metastasis. Notably, hypomethylation at 2 bp upstream of its TSS was reported in diseases such as estrogen and progesterone receptor-negative breast cancers [34], schizophrenia, and bipolar disorder [35]. *SPATS2* was reported to play a critical role in spermatogenesis and development of testicular germ cell [36], and no reports on diseases association except for recent study on, its paralog, *SPATS2L* , in a context of bronchodilator response gene with a genome-wide association study [37]. Further studies are required to elucidate the roles of the novel markers in lung cancer.

Several limitations to using SPATS2 and ST6GALNAC1 as IHC markers in clinical use warrant mention. First, localizations of immunostaining are not limited to the nucleus of tumor cells. IHC staining of only the tumor nucleus is ideal because passive diffusion of non-nuclear markers is observed using small or crushed samples. However, SPATS2 was localized to the cytoplasm of tumor cells but also stained the basal membrane of the alveolar septum and infiltrating plasma cells. ST6GALNAC1 was localized on the cellular membrane of tumor cells but also stained the bronchial epithelium. Second, proportions of score 1 for SPATS2 and ST6GALNAC1 were higher than for other existing IHC markers because the tentative diagnostic criteria for the novel IHC markers were used. Namely, only cases in which more than 50 % of tumor cells showed moderate or more severe immunoreactivity were considered positive, to reduce the rate of false positive results with antibodies not optimized for clinical diagnosis. To our knowledge, no optimized scoring system or optimized antibodies for novel IHC markers using a large number of surgical specimens have been established. Third, this study was performed based on only the surgical specimens. Therefore, further prospective studies based on cytology or small biopsy samples need to be conducted to confirm the utility of novel markers in these clinically meaningful setting.

## Conclusions

We discovered novel biomarkers, ST6GALNAC1 and SPATS2, which assist in accurate discrimination between SCC and AD. We demonstrated that these markers contributed to successful subtyping, even in cases where morphological discrimination was difficult, such as PDSCC and non-lepidic AD. We found that the majority of SCC and AD cases are distinguishable using a combination of ST6GALNAC1 and CK5, while the remaining cases can be distinguished using the combination of TTF-1 and SPATS2. These findings shed light on a new way to accurately subtype NSCLC, contributing to precision medicine for lung cancer.

## Additional file

Additional file 1: Table S1. The immunohistochemical staing conditions and antibodies used in this study. **Table S2**. Evaluation of the markers using the validation set with 45 AD and 29 SCC patients. **Table S3**. Classification of AD and SCC by combinations of AD and SCC markers. **Table S4**. Evaluation of the markers for seven unclassifiable patients showing ST6GALNAC1(-)/CK5(-) or ST6GALNAC1(+)/CK5(+). (DOCX 53 kb)

## Abbreviations

AD: Adenocarcinoma
CAGE: Cap analysis gene expression
CK: Cytokeratin
CPM: Counts per million
DSCC: Differentiated SCC
DSG3: Desmoglein-3
IHC: Immunohistochemical analysis
MD: Moderately differentiated
MDS: Multi-dimensional scaling
NSCLC: Non-small cell lung cancer
PD: Poorly differentiated
SCC: Squamous cell carcinoma
SPATS2: Spermatogenesis associated, serine-rich 2
ST6GALNAC1: ST6 (alpha-N-acetyl-neuraminyl-2,3-beta-galactosyl-1,3)-N-acetylgalactosaminide alpha-2,6-sialyltransferase 1
TSS: Transcription starting site
WD: Well differentiated.
