# Supplementary material for: Novel biomarkers that assist in accurate discrimination of squamous cell carcinoma from adenocarcinoma of the lung
Source: BMC Cancer. 2016 Sep 29;16:760. doi: 10.1186/s12885-016-2792-1 (PMC5041559; doi:10.1186/s12885-016-2792-1)
Supplement: Additional file 1: Table S1. — The immunohistochemical staing conditions and antibodies used in this study. Table S2. Evaluation of the markers using the validation set with 45 AD and 29 SCC patients. Table S3. Classification of AD and SCC by combinations of AD and SCC markers. Table S4. Evaluation of the markers for seven unclassifiable patients showing ST6GALNAC1(-)/CK5(-) or ST6GALNAC1(+)/CK5(+). (DOCX 53 kb) [file 12885_2016_2792_MOESM1_ESM.docx]

Table S1. The immunohistochemical staing conditions and antibodies used in this study

| Antibody | Antigen retrieval | Temperature (°C) | Time (min) | Dilution | Primary Ab reaction time | Secondary Ab reaction time | Clone | Company |
| --- | --- | --- | --- | --- | --- | --- | --- | --- |
| TTF-1 | PH9　TE buffer | 110 | 15 | x75 | O/N at 4°C | Envision  50 min | 8G7G3/1 | DAKO, Carpinteria, CA, USA |
| napsin A | No treatment |  |  | x300 | O/N at 4°C | Envision  45 min | IP64 | Leica Biosystem, Newcastle, NE UK |
| p40 | PH6 citrate buffer | 120 | 10 | x2500 | O/N at 4°C | Envision  45 min | Rabbit poly | Millipore Co., San Diego, CA, USA |
| CK5 | PH6 citrate buffer | 120 | 10 | x200 | O/N at 4°C | Envision  45 min | XM26 | Leica Biosystem, Newcastle, NE UK |
| CK6 | PH9　TE buffer | 100 | 30 | x100 | 2hrs at RT | Envision  45 min | LHK6B | Gene Tex, Irvine, CA, USA |
| DSG3 | PH9　TE buffer | 105 | 30 | x50 | O/N at 4°C after 2 hrs at RT | Envision  75 min | BC11 | BIO CARE Medical, Concord, CA, USA |
| **ST6GALNAC1*** | PH6 citrate buffer | 120 | 10 | x4000 | 90 min at RT | Envision  45 min | Rabbit poly | SIGMA Life Science, St.Louis, MO, USA |
| **SPATS2*** | PH9　TE buffer | 105 | 30 | x50 | 2 hrs at RT | Envision  50 min | Rabbit poly | SIGMA Life Science, St.Louis, MO, USA |

O/N: Overnight; RT: Room temperature.

Table S2. Evaluation of the markers using the validation set with 45 AD and 29 SCC patients

| AD markers | (Marker status) | (+) | |  | (-) | |  | Sensitivity | Specificity | PPV | NPV | Accuracy |
| --- | --- | --- | --- | --- | --- | --- | --- | --- | --- | --- | --- | --- |
|  | (subtype) | AD | SCC |  | AD | SCC |  | (95% CI†) | (95% CI) | (95% CI) | (95% CI) | (95% CI) |
|  | **ST6GALNAC1*** | 43 | 1 |  | 2 | 28 |  | 0.956  (0.849-0.995) | 0.966  (0.822-0.999) | 0.977  (0.880-0.999) | 0.933  (0.779-0.992) | 0.959  (0.886-0.992) |
|  | TTF-1 | 33 | 0 |  | 12 | 29 |  | 0.733  (0.581-0.854) | 1.000  (0.881-1.000) | 1.000  (0.894-1.000) | 0.707  (0.545-0.839) | 0.838  (0.734-0.913) |
|  | napsin A | 35 | 0 |  | 10 | 29 |  | 0.778  (0.629-0.888) | 1.000  (0.881-1.000) | 1.000  (0.900-1.000) | 0.744  (0.579-0.870) | 0.865  (0.765-0.933) |
| SCC markers | (Marker status) | (+) | |  | (-) | |  | Sensitivity | Specificity | PPV | NPV | Accuracy |
|  | (subtype) | SCC | AD |  | SCC | AD |  | (95% CI) | (95% CI) | (95% CI) | (95% CI) | (95% CI) |
|  | **SPATS2*** | 20 | 3 |  | 9 | 42 |  | 0.690  (0.492-0.847) | 0.933  (0.817-0.986) | 0.870  (0.664-0.972) | 0.824  (0.691-0.916) | 0.838  (0.734-0.913) |
|  | CK5 | 25 | 0 |  | 4 | 45 |  | 0.862  (0.683-0.961) | 1.000  (0.921-1.000) | 1.000  (0.863-1.000) | 0.918  (0.804-0.977) | 0.946  (0.867-0.985) |
|  | DSG3 | 24 | 0 |  | 5 | 45 |  | 0.828  (0.642-0.942) | 1.000  (0.921-1.000) | 1.000  (0.858-1.000) | 0.900  (0.782-0.967) | 0.932  (0.849-0.978) |
|  | p40 | 25 | 1 |  | 4 | 44 |  | 0.862  (0.683-0.961) | 0.978  (0.882-0.999) | 0.962  (0.804-0.999) | 0.917  (0.800-0.977) | 0.932  (0.849-0.978) |
|  | CK6 | 23 | 20 |  | 6 | 25 |  | 0.793  (0.603-0.920) | 0.556  (0.400-0.704) | 0.535  (0.377-0.688) | 0.806  (0.625-0.925) | 0.649  (0.529-0.756) |

PPV: Positive predictive value; NPV: Negative predictive value; 95% CI: 95% confidence interval.

†: 95% CIs of sensitivity, specificity, PPV, NPV and accuracy were estimated by the Clopper-Pearson method.

Table S3. Classification of AD and SCC by combinations of AD and SCC markers

| AD Marker | SCC marker | (+) / (-) | |  | (-) / (+) | |  | (+) / (+) | |  | (-) / (-) | |  | % of unclassifiable | Accuracy |
| --- | --- | --- | --- | --- | --- | --- | --- | --- | --- | --- | --- | --- | --- | --- | --- |
|  |  | AD | SCC |  | AD | SCC |  | AD | SCC |  | AD | SCC |  | specimens† |  |
| **ST6GALNAC1*** | **SPATS2*** | 40 | 0 |  | 0 | 19 |  | 3 | 1 |  | 2 | 9 |  | 20.3% | 0.797 |
| **ST6GALNAC1*** | CK5 | 43 | 0 |  | 0 | 24 |  | 0 | 1 |  | 2 | 4 |  | 9.5% | 0.905 |
| **ST6GALNAC1*** | DSG3 | 43 | 0 |  | 0 | 23 |  | 0 | 1 |  | 2 | 5 |  | 10.8% | 0.892 |
| **ST6GALNAC1*** | p40 | 42 | 0 |  | 0 | 24 |  | 1 | 1 |  | 2 | 4 |  | 10.8% | 0.892 |
| **ST6GALNAC1*** | CK6 | 23 | 0 |  | 0 | 22 |  | 20 | 1 |  | 2 | 6 |  | 39.2% | 0.608 |
| TTF-1 | **SPATS2*** | 32 | 0 |  | 2 | 20 |  | 1 | 0 |  | 10 | 9 |  | 27.0% | 0.703 |
| TTF-1 | CK5 | 33 | 0 |  | 0 | 25 |  | 0 | 0 |  | 12 | 4 |  | 21.6% | 0.784 |
| TTF-1 | DSG3 | 33 | 0 |  | 0 | 24 |  | 0 | 0 |  | 12 | 5 |  | 23.0% | 0.770 |
| TTF-1 | p40 | 32 | 0 |  | 0 | 25 |  | 1 | 0 |  | 12 | 4 |  | 23.0% | 0.770 |
| TTF-1 | CK6 | 19 | 0 |  | 6 | 23 |  | 14 | 0 |  | 6 | 6 |  | 35.1% | 0.568 |
| napsin A | **SPATS2*** | 34 | 0 |  | 2 | 20 |  | 1 | 0 |  | 8 | 9 |  | 24.3% | 0.730 |
| napsin A | CK5 | 35 | 0 |  | 0 | 25 |  | 0 | 0 |  | 10 | 4 |  | 18.9% | 0.811 |
| napsin A | DSG3 | 35 | 0 |  | 0 | 24 |  | 0 | 0 |  | 10 | 5 |  | 20.3% | 0.797 |
| napsin A | p40 | 34 | 0 |  | 0 | 25 |  | 1 | 0 |  | 10 | 4 |  | 20.3% | 0.797 |
| napsin A | CK6 | 22 | 0 |  | 7 | 23 |  | 13 | 0 |  | 3 | 6 |  | 29.7% | 0.608 |

†: patients with (+)/(+) or (-)/(-) were defined as unclassifiable.

Table S4. Evaluation of the markers for seven unclassifiable patients showing ST6GALNAC1(-)/CK5(-) or ST6GALNAC1(+)/CK5(+)

| AD markers | (Marker status) | (+) | |  | (-) | |  | Sensitivity | Specificity | PPV | NPV | Accuracy |
| --- | --- | --- | --- | --- | --- | --- | --- | --- | --- | --- | --- | --- |
|  | (subtype) | AD | SCC |  | AD | SCC |  | (95% CI†) | (95% CI) | (95% CI) | (95% CI) | (95% CI) |
|  | TTF-1 | 2 | 0 |  | 0 | 5 |  | 1.000  (0.158-1.000) | 1.000  (0.478-1.000) | 1.000  (0.158-1.000) | 1.000  (0.478-1.000) | 1.000  (0.590-1.000) |
|  | napsin A | 1 | 0 |  | 1 | 5 |  | 0.500  (0.013-0.987) | 1.000  (0.478-1.000) | 1.000  (0.025-1.000) | 0.833  (0.359-0.996) | 0.857  (0.421-0.996) |
| SCC markers | (Marker status) | (+) | |  | (-) | |  | Sensitivity | Specificity | PPV | NPV | Accuracy |
|  | (subtype) | SCC | AD |  | SCC | AD |  | (95% CI) | (95% CI) | (95% CI) | (95% CI) | (95% CI) |
|  | **SPATS2*** | 5 | 0 |  | 0 | 2 |  | 1.000  (0.478-1.000) | 1.000  (0.158-1.000) | 1.000  (0.478-1.000) | 1.000  (0.158-1.000) | 1.000  (0.590-1.000) |
|  | DSG3 | 1 | 0 |  | 4 | 2 |  | 0.200  (0.005-0.716) | 1.000  (0.158-1.000) | 1.000  (0.025-1.000) | 0.333  (0.043-0.777) | 0.429  (0.099-0.816) |
|  | p40 | 1 | 0 |  | 4 | 2 |  | 0.200  (0.005-0.716) | 1.000  (0.158-1.000) | 1.000  (0.025-1.000) | 0.333  (0.043-0.777) | 0.429  (0.099-0.816) |
|  | CK6 | 2 | 0 |  | 3 | 2 |  | 0.400  (0.053-0.853) | 1.000  (0.158-1.000) | 1.000  (0.158-1.000) | 0.400  (0.053-0.853) | 0.571  (0.184-0.901) |

PPV: Positive predictive value; NPV: Negative predictive value; 95% CI: 95% confidence interval.

†: 95% CIs of sensitivity, specificity, PPV, NPV and accuracy were estimated by the Clopper-Pearson method.
